# Bayesian Prediction of Pre-Stressed Concrete Bridge Deflection Using Finite Element Analysis

**DOI:** 10.3390/s19224956

**Published:** 2019-11-14

**Authors:** Jaebeom Lee, Kyoung-Chan Lee, Sung-Han Sim, Junhwa Lee, Young-Joo Lee

**Affiliations:** 1 School of Urban and Environmental Engineering, Ulsan National Institute of Science and Technology (UNIST), Ulsan 44919, Korea; jblee@unist.ac.kr (J.L.); lee.junhwa@unist.ac.kr (J.L.); 2 Advanced Railroad Civil Engineering Division, Korea Railroad Research Institute, Uiwang 16105, Korea; 3 School of Civil, Architectural Engineering and Landscape Architecture, Sungkyunkwan University, Seoul 16419, Korea; ssim@skku.edu

**Keywords:** railway bridge, vertical deflection, probabilistic prediction, Gaussian process regression, finite element

## Abstract

Vertical deflection has been emphasized as an important safety indicator in the management of railway bridges. Therefore, various standards and studies have suggested physics-based models for predicting the time-dependent deflection of railway bridges. However, these approaches may be limited by model errors caused by uncertainties in various factors, such as material properties, creep coefficient, and temperature. This study proposes a new Bayesian method that employs both a finite element model and actual measurement data. To overcome the limitations of an imperfect finite element model and a shortage of data, Gaussian process regression is introduced and modified to consider both, the finite element analysis results and actual measurement data. In addition, the probabilistic prediction model can be updated whenever additional measurement data is available. In this manner, a probabilistic prediction model, that is customized to a target bridge, can be obtained. The proposed method is applied to a pre-stressed concrete railway bridge in the construction stage in the Republic of Korea, as an example of a bridge for which accurate time-dependent deflection is difficult to predict, and measurement data are insufficient. Probabilistic prediction models are successfully derived by applying the proposed method, and the corresponding prediction results agree with the actual measurements, even though the bridge experienced large downward deflections during the construction stage. In addition, the practical uses of the prediction models are discussed.

## 1. Introduction

The vertical deflection of railway bridges is an important safety indicator that is used in the safety management of structures and trains [1,2]. In particular, high-speed trains can be derailed owing to railway defects that can be caused by excessive bridge deflection. Therefore, the monitoring and management of the vertical deflection of railway bridges have been emphasized in several standards, such as the Design Guide for Steel Railway Bridges of the UK (2004), UIC CODE 518 OR (2009), and the Guideline of Track Maintenance of Korea Rail Network Authority (2016), which suggest monitoring for the acceptable vertical deflection at mid-span [3,4,5]. For example, the Korean railway bridge guideline specifies allowable maximum deflections with respect to the span length (*L*) and train velocity. For instance, for train speeds between 200 km/h and 270 km/h, the allowable threshold of vertical deflection with a span length is shorter than 20 m is *L*/1300 m [5].

Pre-stressed concrete (PSC) girders have been widely adopted as superstructures for high-speed railway bridges; the long-term deflection of bridges with PSC girders is known to be dependent on the mechanical responses of concrete, such as creep and shrinkage [6]. Concrete creep and drying shrinkage are time-dependent deformations, caused by sustained loading (e.g., dead load), and loss of moisture, respectively, and these deformations proceed with a decreasing rate for the first several months or years [7]. Considering these factors, a variety of prediction models, such as the CEB-FIP, ACI, KCI, and B3 models, have been developed [8,9,10,11]. However, these generalized prediction models are limited by model errors caused by uncertainties in various factors, such as material properties, creep coefficient, and temperature [12]. Kamatchi et al. (2014) showed that prediction models (i.e., CEB-FIP, ACI, KCI, and B3 models) also involve errors with field measurements [13], and Bažant et al. (2012) revealed that existing material models for predicting creep and shrinkage are unsatisfactory [14,15]. In addition, the deflection prediction of a bridge under construction is more challenging, because the structure experiences sudden loading and unloading during the construction stage. Therefore, it is important to consider the physical phenomena associated with these construction events from an early stage to predict the long-term deflection of a bridge under construction accurately.

Meanwhile, data-driven approaches, which utilize actual measurement data, have been studied to construct prediction models as well. Sun and Hao (2011) introduced a time series analysis method utilizing monitoring data and established a prediction model of bridge deflection [16]. Similarly, Xin et al. (2018) suggested a prediction method for bridge deformation, integrating the Kalman filter, the autoregressive integrated moving average model, and a generalized autoregressive conditional heteroskedasticity process, using various measurement data (i.e., from strain gauges, displacement sensors at mid-span, acceleration sensors, cable meters, and temperature sensors) [17]. A wavelet analysis is introduced in various studies to detect the structural damage [18,19,20,21,22,23], and Lee et al. (2018) introduced a machine-learning-based method to construct a non-parametric prediction model, which showed good agreement with actual measurement data [2].

However, because the amount of measurement data may be insufficient to apply a data-driven approach, a few studies have combined both physics-based models and data-driven approaches. Yang (2007) proposed a method to incorporate measurement data into physics-based prediction models, considering creep and shrinkage to reduce the uncertainty in long-term predictions [24]. Similarly, Bažant et al. (2012) used deflection measurements to update the B3 model [14]. A finite element (FE) model calibration procedure using the monitoring data has often been conducted by researchers studying efficient bridge management [25,26,27,28,29,30].

Inaccurate prediction of bridge deflection may result in unexpected structural failure (e.g., the Koror-Babeldaob bridge failure in 1996); thus, continuous monitoring and management of vertical deflection are important. This study aims to overcome the limitations of the structural model error and data shortage and provide a probabilistic prediction of bridge deflection. To this end, a new Bayesian method is proposed, utilizing an FE model constructed with physics-based knowledge and actual measurement data. The target bridge of this study is a PSC railway bridge for high-speed trains in the construction stage, to which various construction loads are applied. Therefore, an accurate prediction of time-dependent deflection is difficult to obtain, even using a sophisticated FE analysis tool. Moreover, a sufficient quantity of measurement data for predicting the bridge deflection is unavailable because of the short measurement period, and thus, a data-driven approach may be inaccurate. An FE model is constructed by considering the construction schedule and mechanical responses for predicting vertical deflection as prior knowledge. Then, the prediction model, based on physics-based knowledge, is updated by measurement data utilizing Gaussian process regression (GPR), which is a nonparametric Bayesian method. In this manner, the probabilistic interval (e.g., 95% prediction interval) of the expected vertical deflection can be obtained, which can be used for bridge deflection management in the construction stage.

## 2. Gaussian Process Regression (GPR)

### 2.1. Bayesian Inference in GPR

To build a probabilistic prediction model, GPR is used in this study. A Gaussian process (GP) is a stochastic process, by which all data points are assumed to follow a multivariate Gaussian distribution, and implying that the data points are correlated and normally distributed [31]. In GPR, data points are assumed to originate from a GP, called the GP assumption. It is also known that GPR is a Bayesian method that builds a nonparametric regression model using the GP assumption [32].

Let *N_D_* quantities of noisy training dataset **D**, which consists of a training input matrix **X** and a training output vector **y**, be given as,

(1) $$D=\left\{\left(X,y\right)\right\}=\left\{\left(x_{ij},y_{i}\right)|i=1,\ldots ,N_{D};j=1,\ldots ,N_{x}\right\}$$

where *x_ij_* is the element of the training input matrix **X**, whose matrix size is *N_D_* by *N_x_*, and *y_i_* is the element in the training output vector **y**, whose size is *N_D_* by 1. The input-output relationship is expressed as,

(2) y=f(X)+ε=ϕ(X)w+ε

where ϕ(·) is the vector of *N_dim_* projection functions (e.g., ϕ(*z*) = [1, *z*, *z*^2^, *z*^3^] for the three-dimensional polynomial regression of a scalar input *z* or ϕ(*z*) = [1, sin(2π*z*), cos(2π*z*)] for the first-order trigonometric regression), *f*(**X**) is the regression function of **X**, which can be calculated as the product of the projected input matrix ϕ(**X**), and the vector of weights **w** (whose sizes are *N_D_* by *N_dim_* and *N_dim_* by 1, respectively), and **ε** is the vector of observational errors (whose vector size is *N_D_* by 1). Here, the observational errors are assumed to follow an independent and identically distributed Gaussian distribution with a zero-mean and constant variance of the noise, σ*_n_*^2^ for all inputs (i.e., a homoscedastic noise assumption) as:

(3) $$\varepsilon \sim N\left(O,\sigma_{n}^{2}I\right).$$

To infer the weight vector **w** based on actual measurements in the Bayesian view, the prior assumption on the weight vector is set to be a zero-mean Gaussian with the covariance matrix **Σ***_p_*:

(4) $$w\sim N\left(O,\Sigma_{p}\right)$$

which enables the prior prediction on the mean μ_X__*_ and the standard deviation σ_X__*_ based on the given input matrix X_*_ as:

(5) $$f\left(X_{*}\right)=\phi \left(X_{*}\right)w\sim N\left(O,\phi \left(X_{*}\right)\Sigma_{p}\phi {\left(X_{*}\right)}^{T}\right)=N\left(\mu_{X_{*}},\sigma_{X_{*}}^{2}\right).$$

From the independent and identically distributed Gaussian process assumption of observations having homoscedastic noise, the likelihood function can be constructed as the multiplication of normal probability density functions (PDFs) as follows,

(6) $$\begin{array}{l} p\left(y|X,w\right)=\prod_{i=1}^{N_{D}}P\left(y_{i}|\phi \left(x_{i}\right)w\right)=\prod_{i=1}^{N_{D}}\frac{1}{\sqrt{2\pi }\sigma_{n}}\exp\left(-\frac{{\left(y_{i}-\phi \left(x_{i}\right)w\right)}^{2}}{2\sigma_{n}^{2}}\right)\\ =\frac{1}{{\left(2\pi \sigma_{n}^{2}\right)}^{\frac{N_{D}}{2}}}\exp\left(-\frac{1}{2\sigma_{n}^{2}}{\left\|y-\phi \left(X\right)w\right\|}_{2}^{2}\right) \end{array}$$

where *y_i_* is an *i*-th element of the output vector, **x***_i_* is the corresponding input vector, ∥∙∥_2_ is an L2 norm which denotes the Euclidean distance. Consequently, Equation (6) indicates the following relation:

(7) $$y|X,w\sim N\left(\phi \left(X\right)w,\sigma_{n}^{2}I\right).$$

Meanwhile, from the Bayes’ rule, the posterior probability on the weight vector is expressed as:

(8) $$\begin{array}{l} p\left(w|X,y\right)=\frac{p\left(X,y|w\right)p\left(w\right)}{p\left(X,y\right)}=\frac{p\left(y|X,w\right)p\left(X,y\right)}{p\left(y|X\right)}\frac{p\left(w\right)}{p\left(X,y\right)}\\ =\frac{p\left(y|X,w\right)p\left(w\right)}{p\left(y|X\right)}=\frac{p\left(y|X,w\right)p\left(w\right)}{\int p\left(y|X,w\right)p\left(w\right)dw} \end{array}$$

It can be seen from Equation (8) that the posterior probability *p*(**w**|**X**,**y**) is proportional to the prior probability *p*(**w**) and the likelihood *p*(**y**|**X**,**w**), which can be expressed as follows:

(9) p(w|X,y)∝p(y|X,w)p(w).

From Equations (4) and (6), the posterior probability in Equation (9) is seen to be proportional to the multiplication of two multi-variate Gaussian PDFs:

(10) $$p\left(w|X,y\right)\propto \exp\left(-\frac{1}{2\sigma_{n}^{2}}{\left(y-\phi \left(X\right)w\right)}^{T}\left(y-\phi \left(X\right)w\right)\right)\exp\left(-\frac{1}{2}w^{T}\Sigma_{p}^{-1}w\right).$$

That is [31],

(11) $$p\left(w|X,y\right)\propto \exp\left(-\frac{1}{2}{\left[w-\bar{w}\right]}^{T}\left(\sigma_{n}^{-2}\phi {\left(X\right)}^{T}\phi \left(X\right)+\Sigma_{p}^{-1}\right)\left[w-\bar{w}\right]\right)$$

where

(12) $$\bar{w}=\sigma_{n}^{-2}A^{-1}\phi {\left(X\right)}^{T}y$$

and

(13) $$A=\sigma_{n}^{-2}\phi {\left(X\right)}^{T}\phi \left(X\right)+\Sigma_{p}^{-1}.$$

For the given input matrix X_*_, the predictive mean **ϕ**(X_*_)·**w** is [31],

(14) $$\sigma_{n}^{-2}\phi \left(X_{*}\right)A^{-1}\phi {\left(X\right)}^{T}y=\phi \left(X_{*}\right)\Sigma_{p}\phi {\left(X\right)}^{T}{\left(\phi {\left(X\right)}^{T}\Sigma_{p}\phi \left(X\right)+\sigma_{n}^{2}I\right)}^{-1}y$$

and the predictive variance is [31],

(15) $$\begin{array}{l} \phi \left(X_{*}\right)A^{-1}\phi {\left(X_{*}\right)}^{T}=\phi \left(X_{*}\right){\left(\sigma_{n}^{-2}\phi {\left(X\right)}^{T}\phi \left(X\right)+\Sigma_{p}^{-1}\right)}^{-1}\phi {\left(X_{*}\right)}^{T}\\ =\phi \left(X_{*}\right)\Sigma_{p}\phi {\left(X_{*}\right)}^{T}-\phi \left(X_{*}\right)\Sigma_{p}\phi {\left(X\right)}^{T}{\left(\sigma_{n}^{2}+\phi \left(X\right)\Sigma_{p}\phi {\left(X\right)}^{T}\right)}^{-1}\phi \left(X\right)\Sigma_{p}\phi {\left(X_{*}\right)}^{T} \end{array}$$

which is based on the following matric inversion [33]:

(16) $${\left(UWV^{T}+Z\right)}^{-1}=Z^{-1}-Z^{-1}U{\left(W^{-1}+V^{T}Z^{-1}U\right)}^{-1}V^{T}Z^{-1}.$$

In summary, the predictive mean and variance with the posterior inference on the weights vector are:

(17) $$\begin{array}{l} f\left(X_{*}\right)=\phi \left(X_{*}\right)w\\ \sim N\left(\begin{array}{l} \phi \left(X_{*}\right)\Sigma_{p}\phi {\left(X\right)}^{T}{\left(\phi \left(X\right)\Sigma_{p}\phi {\left(X\right)}^{T}+\sigma_{n}^{2}I\right)}^{-1}y,\\ \phi \left(X_{*}\right)\Sigma_{p}\phi {\left(X_{*}\right)}^{T}-\phi \left(X_{*}\right)\Sigma_{p}\phi {\left(X\right)}^{T}{\left(\sigma_{n}^{2}+\phi \left(X\right)\Sigma_{p}\phi {\left(X\right)}^{T}\right)}^{-1}\phi \left(X\right)\Sigma_{p}\phi {\left(X_{*}\right)}^{T} \end{array}\right)\\ =N\left(\mu_{X_{*}},\sigma_{X_{*}}^{2}\right) \end{array}$$

Here, the term ϕ(·)**Σ***_p_*ϕ(·)*^T^* is the covariance matrix of the projected inputs, and when expressed with the capital letter “**K**,” Equation (17) is arranged as:

(18) $$f\left(X_{*}\right)\sim N\left(K_{{}_{*}}{\left(K+\sigma_{n}^{2}I\right)}^{-1}y,K_{**}-K_{*}{\left(\sigma_{n}^{2}+K\right)}^{-1}K_{*}\right)$$

where $K=\phi \left(X\right)\Sigma_{p}\phi {\left(X\right)}^{T}$, $K_{{}_{*}}=\phi \left(X_{*}\right)\Sigma_{p}\phi {\left(X\right)}^{T}$, and $K_{**}=\phi \left(X_{*}\right)\Sigma_{p}\phi {\left(X_{*}\right)}^{T}$.

### 2.2. Covariance Matrix Design Using Kernel Functions

In GPR, the covariance matrix of the projected inputs is numerically estimated using kernel functions. That is, the covariance of two input vectors **X***_a_* and **X***_b_* is defined by the multiplication of two standard deviations and one correlation coefficient, and is expressed with a kernel function *k* as follows,

(19) $$\sigma_{x_{a}}\sigma_{x_{b}}\rho_{x_{a}x_{b}}=k\left(x_{a},x_{b}\right)$$

where $\sigma_{x_{a}}$ and $\sigma_{x_{b}}$ are the standard deviations of **X***_a_*, and **X***_b_*, respectively, and $\rho_{x_{a}x_{b}}$ is the correlation coefficient of **X***_a_* and **X***_b_*. The covariance matrix, based on kernel functions, enables the GP model to fit data even though the data exhibits nonlinearity [31].

If data have the same variance, then the input data are homoscedastic, and the standard deviation must be a constant that is the square root of the homoscedastic variance. Meanwhile, a correlation coefficient between the two stationary data is generally expressed to be inversely proportional to the Euclidean distance for stationary data, and one representative is the squared exponential kernel (*k_SE_*) [31],

(20) $$k_{SE}(x_{a},x_{b})=\theta_{1}^{2}\exp\left(-\frac{1}{2}\frac{{\left\|x_{a}\left(1,i\right),x_{b}\left(1,i\right)\right\|}_{2}^{2}}{\theta_{2}^{2}}\right)$$

where θ_1_^2^ is the variance hyperparameter that controls the vertical scale of the function change, and θ_2_^2^ is the length-scale hyperparameter that controls the horizontal scale of the function change [31].

The variance of noise (σ*_n_*^2^) in Equation (2) is often expressed by a kernel function (*k_var_*) using the Kronecker delta function,

(21) $$k_{var}(x_{a},x_{b})=\theta_{3}^{2}\cdot \delta (x_{a},x_{b})$$

where θ_3_^2^ is the variance hyperparameter for the input noise. In addition, a bias is introduced using a constant kernel (*k_c_*) with the hyperparameter θ_4_:

(22) $$k_{c}(x_{a},x_{b})=\theta_{4}.$$

In this study, a new kernel is suggested by combining the following kernels:

(23) $$k(x_{a},x_{b})=\theta_{1}^{2}\exp\left(-\frac{1}{2}\frac{{\left\|x_{a}\left(1,i\right),x_{b}\left(1,i\right)\right\|}_{2}^{2}}{\theta_{2}^{2}}\right)+\theta_{3}^{2}\cdot \delta (x_{a},x_{b})+\theta_{4}.$$

These hyperparameters strongly affect the performance of GPR, and their optimal values are estimated based on the given observation data and updated whenever additional data is available. For this purpose, the marginal likelihood *p*(**y**|**X**), which means the probability to observe the output **y** given the input **X**, is introduced. The marginal likelihood of the observations with the prior inference on the weights vector is expressed as the probability density function of the multivariate Gaussian distribution as follows:

(24) $$p(y|X)=\frac{1}{{\left(2\pi \right)}^{\frac{N_{D}}{2}}\cdot {\left|K+\sigma_{n}^{2}I\right|}^{\frac{1}{2}}}\cdot \exp\left(-\frac{1}{2}y^{T}{\left(K+\sigma_{n}^{2}I\right)}^{-1}y\right).$$

Then, the marginal likelihood will be maximized with the best combination of the hyperparameters, which yields an optimization problem. In this study, for numerical convenience, the minus log-marginal likelihood is utilized as the objective function (*L*):

(25) $$L=-\ln p(y|X)=\frac{1}{2}y^{T}{\left(K+\sigma_{n}^{2}I\right)}^{-1}y+\frac{1}{2}\ln\left|K+\sigma_{n}^{2}I\right|+\frac{N_{D}}{2}\ln\left(2\pi \right).$$

The best hyperparameters (**θ***_best_*) can be determined through optimization to minimize the objective function:

(26) $$\theta_{best}=\underset{\theta }{\arg\min}(L).$$

### 2.3. GPR with FE Analysis Results

In several previous studies, the prior mean function in GPR is set to a zero matrix, as described in Equation (4). However, the results of FE analysis can be introduced as the prior mean *m*(∙), also called the explicit basis function, and the input-output relationship is expressed as:

(27) y=f(X)+ε={m(X)+ϕ(X)w}+ε.

Compared with Equation (2), Equation (27) is seen to include the prior mean based on FE analysis, and this can help to overcome the limitation on predictions of the vertical deflection of a bridge under construction, when sufficient data is not available.

For computational simplicity, the residual differences between the output values and the explicit basis function values are considered as new outputs as follows,

(28) $$y^{\prime }=y-m\left(X\right)=f^{\prime }\left(X\right)+\varepsilon =\phi \left(X\right)w+\varepsilon$$

where **y**′ is the set of new training outputs, and *f*’(·) is the new regression function. Then, with the new training outputs (**y**′), we have an input-output relationship with a zero prior mean again, which was discussed in Section 2.1. The marginal likelihood can now be expressed as the conditional probability of the new training outputs given the training input matrix (**X**) as follows:

(29) $$p(y^{\prime }|X)=\frac{1}{{\left(2\pi \right)}^{\frac{N_{D}}{2}}\cdot {\left|K+\sigma_{n}^{2}I\right|}^{\frac{1}{2}}}\cdot \exp\left(-\frac{1}{2}y^{\prime T}{\left(K+\sigma_{n}^{2}I\right)}^{-1}y^{\prime }\right).$$

Once the hyperparameters are optimized, the predictive outputs can be estimated as follows,

(30) $$f^{\prime }\left(X_{*}\right)\sim N\left({Κ}_{*}^{T}{\left(Κ+\sigma_{n}^{2}I\right)}^{-1}y^{\prime },\;\;K_{**}-K_{*}^{T}{\left(K+\sigma_{n}^{2}I\right)}^{-1}K_{*}\right)$$

which is equivalent to:

(31) $$f\left(X_{*}\right)\sim N\left(m\left(X_{*}\right)+{Κ}_{*}^{T}{\left(Κ+\sigma_{ne}^{2}I\right)}^{-1}\left(y-m\left(X\right)\right),\;K_{**}-K_{*}^{T}{\left(K+\sigma_{n}^{2}I\right)}^{-1}K_{*}\right).$$

### 2.4. Approximation Algorithm for Reducing Computational Cost

One of the limitations involved in the application of the GPR is the computational cost. GPR requires the calculation of the inverse matrix of the covariance matrix, as explained in Section 2.1; hence, the cost of GPR is sensitive to the size of the input and output datasets. To address this issue, various approximation methods were developed, which use a partial dataset to derive performance that is comparable to a standard GPR, where a full dataset is applied [31,34]. Among these methods, the Subset of Data (SoD) approximation, which is simple and known to have a better speed-accuracy trade-off than other approximations [35,36], is introduced for this study.

The key idea of the SoD approximation is to select the subset that provides a reasonable hyperparameter optimization, compared with the regression, using the whole dataset. Utilizing only the *M_D_* number among the *N_D_* quantities of the full dataset (*M_D_* < *N_D_*) naturally decreases the computational cost. The important task here is to select subset points, which facilitate an acceptable regression performance for building the posterior distribution, called the active set. Data selection algorithms, which use an active learning criterion (e.g., differential entropy [37], matching pursuit [38], or information gain [39]) have been used as more effective approaches because randomly selected subset points generally do not perform well [31,36]. This study introduces the differential entropy score suggested by Lawrence et al. (2003) as the criterion, which is maximized at the point with the largest variance [37]. Using the selection criterion, a portion of the whole dataset is selected as the active set (**D***_A_*),

(32) $$D_{A}=\left\{\left(X_{A},y_{A}\right)\right\}=\left\{\left(x_{ij},y_{i}\right)|i=1,\ldots ,M_{D};j=1,\ldots ,N_{x}\right\}$$

where **X***_A_* and **y***_A_* are the selected training input matrix, and output vector (i.e., active set), respectively, whose sizes are *M_D_* by *N_x_* and *M_D_* by 1. Once the active set is selected, the hyperparameters are optimized by utilizing the maximum likelihood concept described in Equations (29)–(31) with the active set. The marginal likelihood of the training output vector given the training input matrix of the active set can be described as follows,

(33) $$p(y_{A}|X_{A})=\frac{1}{{\left(2\pi \right)}^{\frac{M_{D}}{2}}\cdot {\left|K_{A}+\sigma_{n}^{2}I\right|}^{\frac{1}{2}}}\cdot \exp\left(-\frac{1}{2}y_{A}{}^{T}{\left(K_{A}+\sigma_{n}^{2}I\right)}^{-1}y_{A}\right)$$

where **K***_A_* is the covariance matrix of the training input matrix of the active set **X***_A_*, whose size is *M_D_* by *M_D_*. The computational cost for calculating the inverse matrix in the likelihood function is much lower because of the small size of the covariance matrix. After optimizing the hyperparameters, the predictive outputs can be estimated:

(34) $$f\left(X_{*}\right)\sim N\left(K_{A*}{\left(K_{A}+\sigma_{n}^{2}I\right)}^{-1}y,K_{A**}-K_{A*}{\left(\sigma_{n}^{2}+K_{A}\right)}^{-1}K_{A*}\right)$$

where **K**_A__*_ is the covariance matrix between the selected training (**X***_A_*) inputs and the *N_P_* number of prediction (X_*_) inputs, and **K**_A__**_ is the covariance matrix of the prediction input matrix X_*_, whose sizes are *M_D_* by *N_P_* and *N_P_* by *N_P_*, respectively. When the explicit basis function is incorporated as the prior mean, the new outputs are calculated as:

(35) $$f\left(X_{*}\right)\sim N\left(\begin{array}{l} m\left(X_{*}\right)+{Κ}_{A*}^{T}{\left({Κ}_{A}+\sigma_{n}^{2}I\right)}^{-1}\left(y_{A}-m\left(X_{A}\right)\right),\\ K_{A**}-K_{A*}^{T}{\left(K_{A}+\sigma_{n}^{2}I\right)}^{-1}K_{A*} \end{array}\right).$$

## 3. Application Example

### 3.1. Example Bridge Description

**Figure 1:**
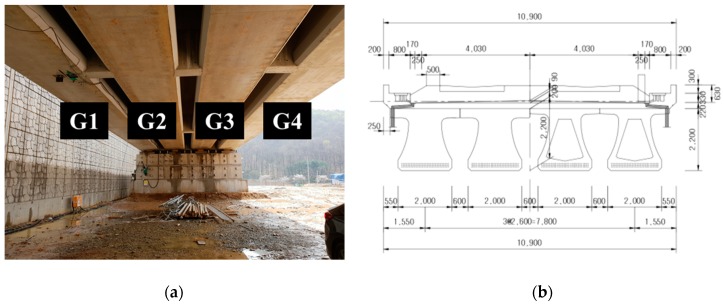
(**a**) Four pre-stressed concrete (PSC) girders of the example bridge; (**b**) a cross-sectional drawing.

To test the proposed method, it was applied to a full-scale railway bridge under construction. The bridge consists of four PSC girders (G1, G2, G3, and G4, as shown in Figure 1a), and each girder has a trivet-shaped cross section, as shown in Figure 1b, and a width, height, and length of 2.0 m, 2.2 m, and 39.94 m, respectively. Among these four girders, G1 was selected as the target, for which actual measurements were taken, by considering various physical properties, such as vertical deflection and temperature.

**Figure 2:**
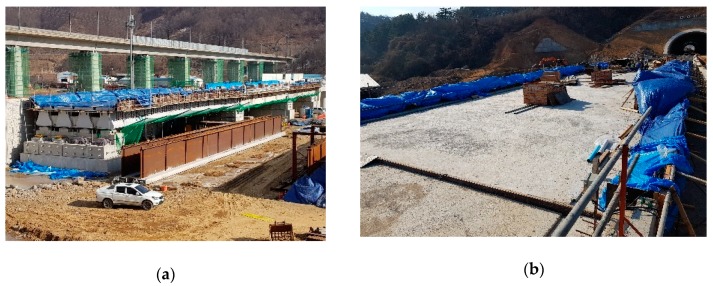
Bridge photos in the construction stages of (**a**) slab placement; and (**b**) protective wall placement.

By following the schedule listed in Table 1, the girder experienced construction loads by the tensioning and detensioning of tendons and substructure placements. The bridge construction started with girder concrete casting and tendon tensioning, which caused an upward deflection on 18 September 2017, defined as day index 1. Tendon releasing and girder erection proceeded in sequence on 22 September (day index 5), and 6 November (day index 50), respectively. Figure 2a shows the slab placement, which was performed from 25 January to 8 February 2018, and it caused a downward deflection. Figure 2b shows the placement of protective walls, which was performed from 14 February to 16 March 2018, and it caused additional downward deflection of the target bridge girder.

### 3.2. Prior prediction based on FE analysis

**Figure 3:**
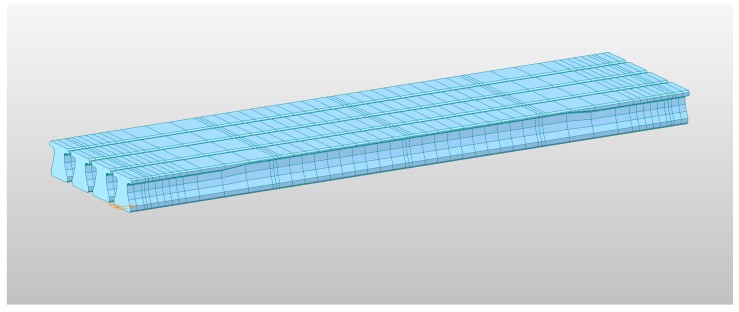
Finite element (FE) model of the example bridge for MIDAS/CIVIL 2017.

For the prior prediction of the time-dependent vertical deflection of the target bridge, in this study, the three-dimensional FE model, established by Lee et al. (2018) is introduced [40]. The FE model was constructed using commercial FE analysis software, MIDAS/CIVIL 2017, which supports the consideration of a construction schedule and the time-dependent structural responses of creep and shrinkage [40,41]. The FE model has 212 nodes and 208 elements, and the constructed model is shown in Figure 3. In addition, the material properties of concrete and steel strands are summarized in Table 2 and Table 3, respectively. The concrete for the slab, cross beam, and girder were modeled using the *C30*, *C30*, and *C49* models of MIDAS/CIVIL 2017, respectively, and the *SWPC7B 15.2 mm, Low Relaxation* model of MIDAS/CIVIL 2017 was used for strands. The construction stages and the associated schedule in Table 1 were considered by conducting a *construction stage analysis* in the software, and the KCI-USD12 code [10] was utilized to estimate the time-dependent deflection of the example bridge. The FE analysis result for the vertical deflection of the bridge, during the construction stages, was introduced as the explicit basis function in the GPR, and the prior prediction was updated using actual measurement data.

### 3.3. Measurement Data

**Figure 4:**
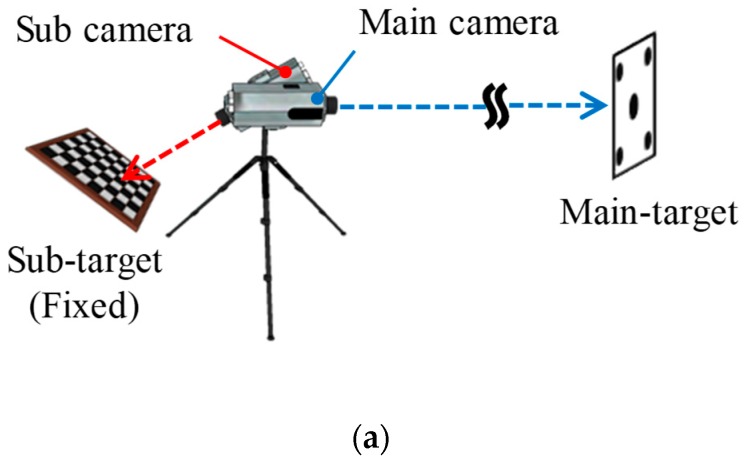

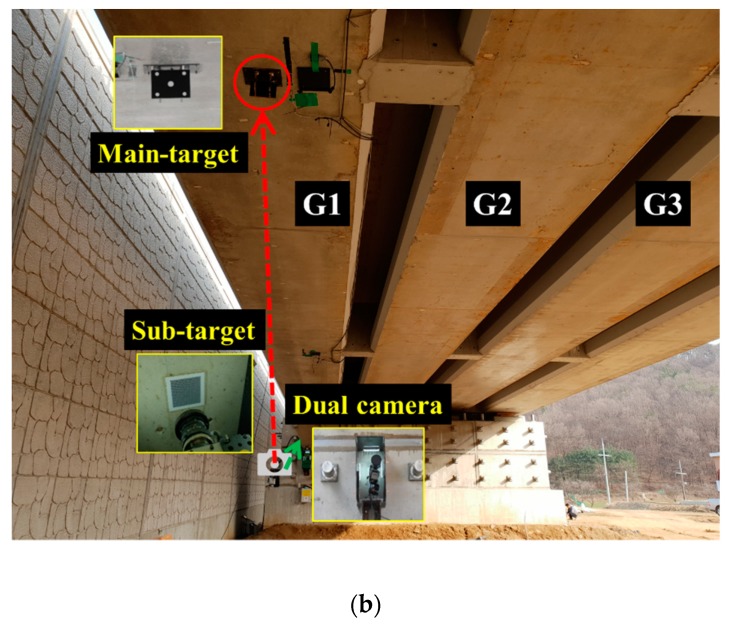
Experimental setup: (**a**) Dual camera configuration; (**b**) deployment of the computer vision system and the thermometer.

The vertical deflection was measured using the computer vision system proposed by Lee et al. [42]. A vision system with the hardware configurations shown in Figure 4a was deployed to monitor the displacement of the main target. In this dual-camera system, the displacement of the main target can be measured, regardless of camera movement, by placing a sub-target displacement at a stationary reference point. As shown in Figure 4b, the main target was deployed at the mid-span of the girder at which displacement was monitored. With the specifications of the camera and the lens listed in Table 4, the employed computer vision system can measure displacement with a resolution of 0.2 mm at a distance of 20 m.

**Figure 5:**
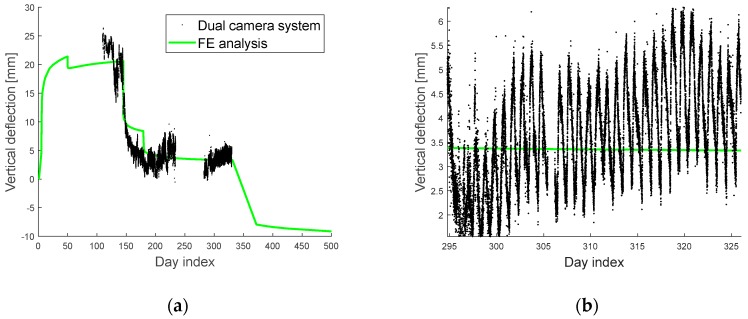
Plots of (**a**) sensor data of vertical deflection versus the prior prediction from the FE analysis; (**b**) a magnified view of a portion of the plot in Figure 5(**a**).

A total of 34,468 measurements were obtained for about nine months, beginning on 4 January 2018. Figure 5a shows the measurement data and the FE analysis results for the vertical deflection. Due to a malfunction in the dual-camera system, sensor data were not obtained from 10 May (day index 235) to 25 June (day index 281) 2018. The FE analysis results (represented by the green line) considers the construction schedule and the associated physical phenomena, and thus, it follows the trend of the measurements by the dual-camera system represented by black dots. However, the FE analysis can provide only a long-term deterministic prediction, without daily fluctuations, as shown in Figure 5b, which is a magnification of a portion of the plot in Figure 5a. Moreover, it is observed that the FE analysis results have a bias. That is, the FE analysis result cannot exactly predict the vertical deflection alone due to its model error.

### 3.4. Probabilistic Prediction Results

**Figure 6:**
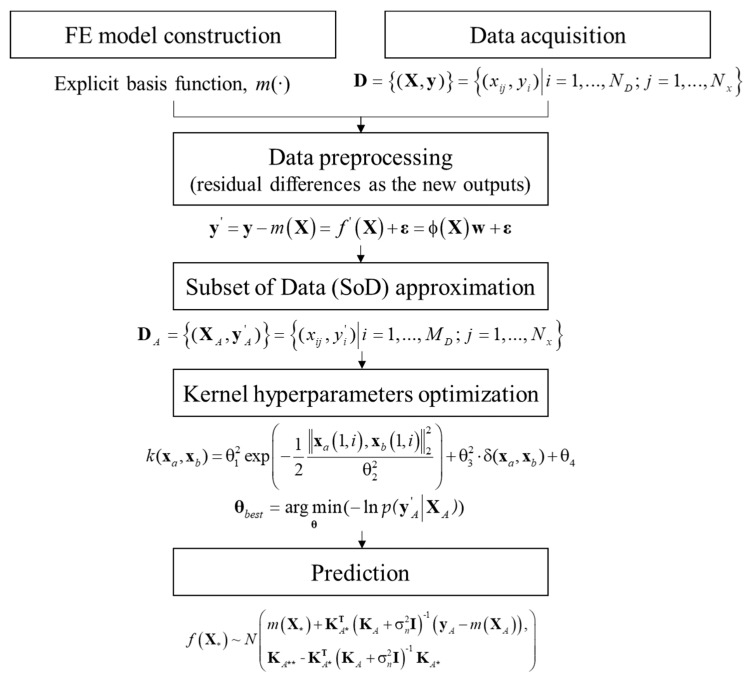
Flow chart of the probabilistic prediction procedure.

**Figure 7:**
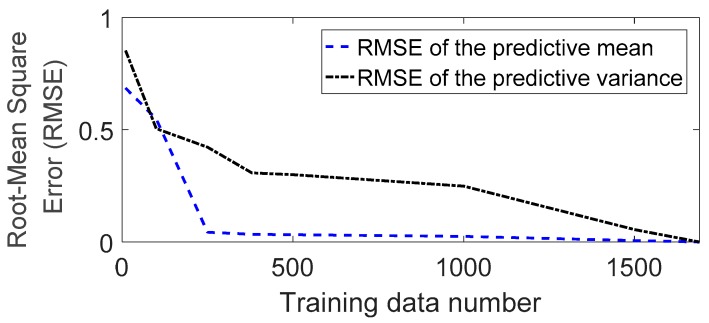
Convergence test result for the training data number using the SoD approximation.

The proposed method is applied to build probabilistic prediction models, and the flow chart, shown in Figure 6, summarizes the procedure. First, data are processed with the measurement data according to the FE model. Second, the SoD approximation is applied to reduce the computational cost. In this application example, a convergence test of the SoD approximation was conducted for the full set of 1690 data, and Figure 7 shows the results. It is observed from the figure that the root mean square errors (RMSEs) of predictive mean and variance decrease with the increasing amount of considered data. Based on these results, the minimum number of SoD was selected to be 400. Next, kernel hyperparameters were determined through an optimization procedure using SoD, and the predictive mean and variance were calculated.

**Figure 8:**
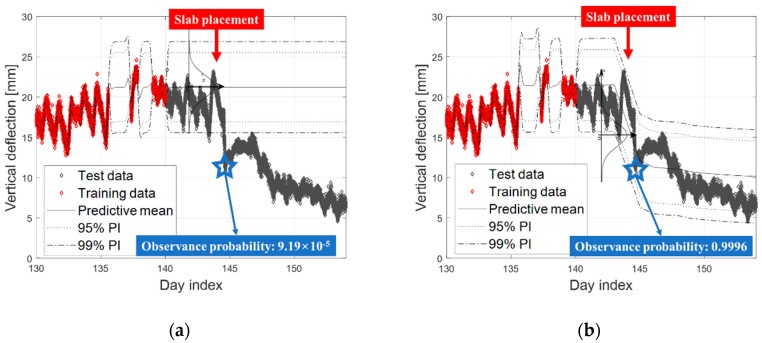
Probabilistic prediction model trained using the data before the slab placement (**a**) without consideration of the FE analysis result; and (**b**) with the result.

The measurement data from day index 109 to 140 is used, and the prediction results with, and without, the use of the FE analysis results are presented in Figure 8a,b respectively. It is seen in both of the figures that, until day 145, the actual measurement data on the vertical deflection are primarily located within the 99% prediction interval (PI). In Figure 8a, however, the measurement data are outside the prediction interval on day 145, when the slab was placed. This is because the slab placement produced a large downward deflection on the target girder, and it could not be considered without the FE analysis. As a result, the predictive mean and standard deviation of the vertical deflection are estimated to be 20.849, and 2.099 mm, respectively. However, the actual measurement on day 145 was 12.64 mm. Using the normal distribution assumption on the vertical deflection in GPR, the probability of observing at least the level of deviation from the mean is calculated to be 2×Φ(−|12.64−20.849|/2.099) = 9.19 × 10^−5^, where Φ(∙) is the standard normal cumulative distribution function (CDF), which indicates a very rare event. On the other hand, when the FE analysis result is used for the probability prediction, the predictive mean and standard deviation are estimated to be 12.639 and 2.364 mm for day 145, and the probability of observing at least the level of deviation from the mean is calculated to be 2×Φ(−|12.64−12.639|/2.364) = 0.9996, as shown in Figure 8b, which indicates a very probable event. These probabilities can be used for effective bridge management.

**Figure 9:**
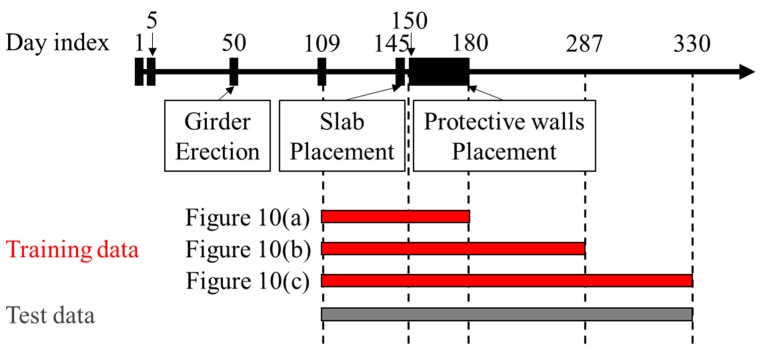
Construction schedule and the training and test data of each prediction model.

**Figure 10:**
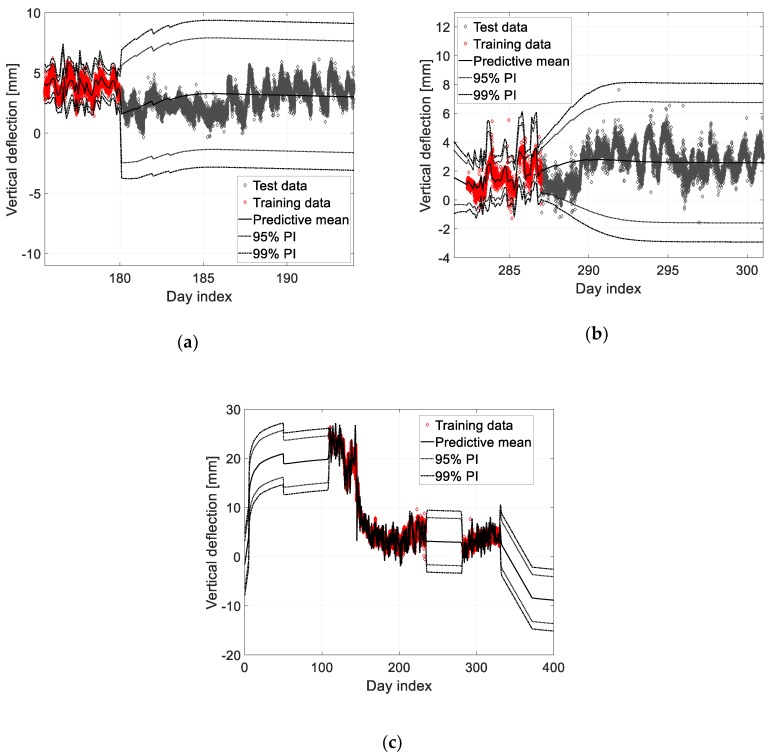
Probabilistic prediction model trained using the data between day indices (**a**) 109 and 180; (**b**) 109 and 287; (**c**) 109 and 330.

Similarly, predictions are made using measurement data for longer periods, as described in Figure 9, by employing both, the measurement data and the prior prediction from the FE analysis. According to a previous study [2], measurement data, obtained for approximately three months, were necessary for an accurate prediction on bridge deflection when considering environmental effects. If sufficient training data are not available, environmental effects can be considered in the prediction through their introduction into the FE analysis. However, this is not within the scope of this study. The prediction model in Figure 10a is constructed using the measurement data between the day indices 109 and 180, and the model in Figure 10b utilized the data between the day indices 109 and 287. First, Figure 10a, which describes the deflection after the placement of the protective walls shows that the vertical deflection after the protective wall placement is within the expected range. Similarly, Figure 10b shows that the deflections are within the acceptable ranges, which means that the construction has proceeded as expected. Finally, the long-term deflection prediction model was constructed using the entire measurements from day indices 109 to 330, as shown in Figure 10c.

As described above, the proposed method can be utilized to calculate the observance probability of an actual measurement. If the observance probability is estimated to be low, it may indicate that there is a serious problem with the bridge, and the bridge manager may consider shutting down the bridge immediately. On the other hand, if the observance probability is estimated to be high, despite the large deflection, the bridge manager can be confident that there is no problem with the bridge. In this manner, the proposed method is expected to support the efficient monitoring and management of bridges.

## 4. Conclusions

This study suggested a new nonparametric Bayesian method, which employs both an FE model and actual measurement data to construct the probabilistic prediction model of bridge deflection. To overcome the limitations of an imperfect FE model and a shortage of data, the GPR was introduced and modified to consider both, the FE analysis results and actual measurement data. In addition, the probabilistic prediction model can be updated whenever additional measurement data is available. The proposed method was applied to an actual railway bridge in the Republic of Korea, which is a PSC bridge under construction, and the probabilistic prediction results agreed with the measurements. In addition, the proposed method calculates the observance probability for a given measurement, which can be useful for efficient monitoring and management of bridges.

## Figures and Tables

**Table 1:** Construction schedule of the example bridge.

Date	Day Index	Construction Schedule
18 September 2017	1	Girder concrete casting and tendon tensioning
22 September 2017	5	Tendon release
6 November 2017	50	Girder erection
8–9 February 2018	144–145	Slab placement
14 February 2018–16 March 2018	150–180	Protective wall placement

**Table 2:** Material properties of concrete.

Classification	Concrete		
	Slab	Cross Beam	Girder
Material code in MIDAS	*C30*	*C30*	*C49*
Compressive strength	30 MPa	30 MPa	49 MPa
Modulus of elasticity	27.52 GPa	27.52 GPa	32.08 GPa
Unit weight	2.5 ton/m^3^	2.5 ton/m^3^	2.5 ton/m^3^
Poisson’s ratio	0.18	0.18	0.18

**Table 3:** Material properties of strand.

Classification	Strand
Material code in MIDAS	*SWPC7B 15.2 mm, Low Relaxation*
Modulus of elasticity	200,000 MPa
Unit weight	7.85 ton/m^3^
Poisson’s ratio	0.3
Total tendon area	15.2 mm × 90 EA
Ultimate strength	1900 MPa
Yield strength	1600 MPa

**Table 4:** Hardware specifications.

Item	Specifications
Main camera	Telescope lens (focal length: 800 mm) 1920 × 1200 resolution
Sub-camera	Wide-angle lens (focal length: 6 mm) 1920 × 1200 resolution
Main target	Five circular dots 15 × 11 cm^2^
Sub-target	32 × 31 checkerboard

## References

[1] Guo T., Sause R., Frangopol D.M., Li A. (2010). Time-dependent reliability of PSC box-girder bridge considering creep, shrinkage, and corrosion. J. Bridge Eng..

[2] Lee J., Lee K.C., Lee Y.J. (2018). Long-Term Deflection Prediction from Computer Vision-Measured Data History for High-Speed Railway Bridge. Sensors.

[3] International Union of Railways (UIC) (2009). Testing and Approval of Railway Vehicle from the Point of View of Their Dynamic Behavior-Safety-Track Fatigue-Running behavior.

[4] Iles D.C. (2004). Design Guide for Steel Railway Bridges.

[5] Korea Rail Network Authority (2016). Guideline of Track Maintenance.

[6] Guo T., Liu T., Li A. (2012). Deflection reliability analysis of PSC box-girder bridge under high-speed railway loads. Adv. Struct. Eng..

[7] Nilson A.H., Winter G., Urquhart L.C., Charles Edward O.R. (1991). Design of Concrete Structures.

[8] Fédération Internationale Du Béton (1999). Structural Concrete: Textbook on Behaviour, Design and Performance.

[9] Videla C., Carreira D.J., Garner N. (2008). Guide for modeling and calculating shrinkage and creep in hardened concrete. ACI Rep..

[10] KCI Committee (2012). Concrete Design Code and Commentary.

[11] Bažant Z.P., Baweja S. (2000). Creep and shrinkage prediction model for analysis and design of concrete structures: Model B3. ACI Spec. Publ..

[12] Bažant Z.P., Yu Q., Li G.H., Klein G.J., Kristek V. (2010). Excessive deflections of record-span prestressed box girder. Concr. Int..

[13] Kamatchi P., Rao K.B., Dhayalini B., Saibabu S., Parivallal S., Ravisankar K., Iyer N.R. (2014). Long-term prestress loss and camber of box-girder bridge. ACI Struct. J..

[14] Bažant Z.P., Yu Q., Li G.H. (2012). Excessive long-time deflections of prestressed box girders. I: Record-span bridge in Palau and other paradigms. J. Struct. Eng..

[15] Bažant Z.P., Yu Q., Li G.H. (2012). Excessive long-time deflections of prestressed box girders. II: Numerical analysis and lessons learned. J. Struct. Eng..

[16] Sun L., Hao X.W. (2011). Analysis of Bridge Deflection Based on Time Series. Appl. Mech. Mater..

[17] Xin J., Zhou J., Yang S., Li X., Wang Y. (2018). Bridge structure deformation prediction based on GNSS data using Kalman-ARIMA-GARCH model. Sensors.

[18] Pnevmatikos N.G., Hatzigeorgiou G.D. (2017). Damage detection of framed structures subjected to earthquake excitation using discrete wavelet analysis. Bull. Earthq. Eng..

[19] Pnevmatikos N.G., Blachowski B., Hatzigeorgiou G.D., Swiercz A. (2016). Wavelet analysis based damage localization in steel frames with bolted connections. Smart Struct. Syst..

[20] Liu J.L., Wang Z.C., Ren W.X., Li X.X. (2015). Structural time-varying damage detection using synchrosqueezing wavelet transform. Smart Struct. Syst..

[21] Wang C., Ren W.X., Wang Z.C., Zhu H.P. (2014). Time-varying physical parameter identification of shear type structures based on discrete wavelet transform. Smart Struct. Syst..

[22] Law S.S., Zhu X.Q., Tian Y.J., Li X.Y., Wu S.Q. (2013). Statistical damage classification method based on wavelet packet analysis. Struct. Eng. Mech..

[23] Fan Z., Feng X., Zhou J. (2013). A novel transmissibility concept based on wavelet transform for structural damage detection. Smart Struct. Syst..

[24] Yang I.H. (2007). Prediction of time-dependent effects in concrete structures using early measurement data. Eng. Struct..

[25] Guo T., Chen Z. (2016). Deflection control of long-span PSC box-girder bridge based on field monitoring and probabilistic FEA. J. Perform. Constr. Facil..

[26] Lee Y.J., Kim R., Suh W., Park K. (2017). Probabilistic fatigue life updating for railway bridges based on local inspection and repair. Sensors.

[27] Kim R.E., Moreu F., Spencer B.F. (2015). System identification of an in-service railroad bridge using wireless smart sensors. Smart Struct. Syst..

[28] Moreu F., Kim R.E., Spencer B.F. (2017). Railroad bridge monitoring using wireless smart sensors. Struct. Control Health Monit..

[29] Kim R.E., Moreu F., Spencer B.F. (2016). Hybrid model for railroad bridge dynamics. J. Struct. Eng..

[30] He W.Y., Zhu S. (2015). Adaptive-scale damage detection strategy for plate structures based on wavelet finite element model. Struct. Eng. Mech..

[31] Rasmussen C.E., Williams C.K. (2006). Gaussian Processes for Machine Learning.

[32] Barber D. (2012). Bayesian Reasoning and Machine Learning.

[33] Hager W.W. (1989). Updating the inverse of a matrix. SIAM Rev..

[34] Snelson E.L. (2007). Flexible and Efficient Gaussian Process Models for Machine Learning. Ph.D. Thesis.

[35] Chalupka K., Williams C.K., Murray I. (2013). A framework for evaluating approximation methods for Gaussian process regression. J. Mach. Learn. Res..

[36] Liu H., Ong Y.S., Shen X., Cai J. (2018). When Gaussian process meets big data: A review of scalable GPs. arXiv.

[37] Herbrich R., Lawrence N.D., Seeger M. (2002). Fast sparse Gaussian process methods: The informative vector machine. Proceedings of the 15th International Conference on Neural Information Processing Systems.

[38] Keerthi S., Chu W. (2006). A matching pursuit approach to sparse gaussian process regression. Proceedings of the 18th International Conference on Neural Information Processing Systems.

[39] Seeger M. (2003). Bayesian Gaussian Process Models: PAC-Bayesian Generalisation Error Bounds and Sparse Approximations.

[40] Lee J., Lee K.C., Kwon H.C., Lim K.M., Min K.H. (2018). Long-term Behavior of Pretension Girder Bridges by Construction Phase Time. J. Korean Soc. Hazard Mitig..

[41] (2017). Midas Civil 2017 [Computer Software].

[42] Lee J., Lee K.C., Jeong S., Lee Y.J., Sim S.H. (2019). Long-term displacement of full-scale bridges using camera ego-motion compensation. Mechanical Systems and Signal Processing, Under Review.

